# Sex-related differences in the response of anti-platelet drug therapies targeting purinergic signaling pathways in sepsis

**DOI:** 10.3389/fimmu.2022.1015577

**Published:** 2022-11-02

**Authors:** Emmanuel Boadi Amoafo, Philomena Entsie, Samara Albayati, Glenn P. Dorsam, Satya P. Kunapuli, Laurie E. Kilpatrick, Elisabetta Liverani

**Affiliations:** ^1^ Department of Pharmaceutical Sciences, School of Pharmacy, College of Health Professions, North Dakota State University, Fargo, ND, United States; ^2^ Sol Sherry Thrombosis Research Center, Temple University School of Medicine, Temple University Hospital, Philadelphia, PA, United States; ^3^ Center for Inflammation and Lung Research, Department of Microbiology, Immunology and Inflammation, Lewis Katz School of Medicine, Temple University, Philadelphia, PA, United States; ^4^ Department of Microbiological Sciences, College of Agriculture, Food Systems and Natural Resources, North Dakota State University, Fargo, ND, United States

**Keywords:** platelets (PLT), purinergic signaling, sex-related differences, sepsis, platelet activation

## Abstract

Sepsis, a complex clinical syndrome resulting from a serious infection, is a major healthcare problem associated with high mortality. Sex-related differences in the immune response to sepsis have been proposed but the mechanism is still unknown. Purinergic signaling is a sex-specific regulatory mechanism in immune cell physiology. Our studies have shown that blocking the ADP-receptor P2Y_12_ but not P2Y_1_ receptor was protective in male mice during sepsis, but not female. We now hypothesize that there are sex-related differences in modulating P2Y_12_ or P2Y_1_ signaling pathways during sepsis. Male and female wild-type (WT), P2Y_12_ knock-out (KO), and P2Y_1_ KO mice underwent sham surgery or cecal ligation and puncture (CLP) to induce sepsis. The P2Y_12_ antagonist ticagrelor or the P2Y_1_ antagonist MRS2279 were administered intra-peritoneally after surgery to septic male and female mice. Blood, lungs and kidneys were collected 24 hours post-surgery. Sepsis-induced changes in platelet activation, secretion and platelet interaction with immune cells were measured by flow cytometry. Neutrophil infiltration in the lung and kidney was determined by a myeloperoxidase (MPO) colorimetric assay kit. Sepsis-induced platelet activation, secretion and aggregate formation were reduced in male CLP P2Y_12_ KO and in female CLP P2Y_1_ KO mice compared with their CLP WT counterpart. Sepsis-induced MPO activity was reduced in male CLP P2Y_12_ KO and CLP P2Y_1_ KO female mice. CLP males treated with ticagrelor or MRS2279 showed a decrease in sepsis-induced MPO levels in lung and kidneys, aggregate formation, and platelet activation as compared to untreated male CLP mice. There were no differences in platelet activation, aggregate formation, and neutrophil infiltration in lung and kidney between female CLP mice and female CLP mice treated with ticagrelor or MRS2279. In human T lymphocytes, blocking P2Y_1_ or P2Y_12_ alters cell growth and secretion *in vitro* in a sex-dependent manner, supporting the data obtained in mice. In conclusion, targeting purinergic signaling represents a promising therapy for sepsis but drug targeting purinergic signaling is sex-specific and needs to be investigated to determine sex-related targeted therapies in sepsis.

## Introduction

Sepsis, a complex clinical syndrome resulting from a serious infection, is a major healthcare problem associated with high morbidity and mortality (1). Current sepsis treatments are limited to supportive therapies (2), and specific pharmacologic treatments that could greatly improve patient outcomes have not yet been developed (2, 3). With hospital mortality rates of affected patients reportedly as high as 50%, there is a critical need for improved methods for treating sepsis (1).

Sex-related differences in the morbidity and mortality of sepsis have been observed in animal models and human diseases (4–8). To date, females has shown decreased mortality and organ failure in mice and humans compared to their male counterpart (4, 7). However, the lack of studies comparing both sexes limit our capacity to evaluate the extent of sex-related differences. Hence sex should be taken into account when identifying the optimal pharmacological intervention for sepsis. Sex-related differences have been observed in other diseases, such as cardiovascular diseases (4), Alzheimer’s (9), cancer (10), and ulcerative colitis (11). In some cases, such as ulcerative colitis (11) and cardiovascular diseases (4) sex-specific treatment has been identified, improving the patient outcome. But there is a gap in knowledge for other diseases.

Purinergic signaling represents a novel regulatory mechanism in immune cell physiology (12). Cells respond to activation with the release of cellular ATP, which regulates cell functions (13). In sepsis, large amounts of ADP are released by tissue damage and immune cells. This leads to over-activation of purinergic signaling contributing to immune dysfunction (13). As a result, regulating purinergic signaling can reveal new avenues in the treatment of sepsis.

Changes in purinergic receptors has been investigated within sexes. For instance, changes in purinergic signaling response may be hormone-dependent (14–17) and they vary in the sex organs (18–20). Indeed, in a murine menopause model, ovarian protein levels of purinergic receptor P2X2 increased with ageing, suggesting that the P2X2 receptor is involved with menopause/ageing-related decline in ovarian function in females (21). Beaucage and coworkers revealed that the P2X7 receptor subtype might be involved in an age- and sex-dependent regulation of adipogenesis and lipid metabolism (14, 16).

Platelet ADP-induced activation is regulated by the P2Y_12_ and P2Y_1_ signaling pathways (22, 23). P2Y_12_ is a G_i_ protein coupled receptor (24), while P2Y_1_ is a G_q_ protein coupled receptor (23, 24). Activation of the P2Y_12_ signaling pathway leads to platelet aggregation and potentiation of platelet secretion (25) while P2Y_1_ activation leads to shape change and aggregation (23, 24). We have previously shown that blockade of the P2Y_12_ signaling pathway in a murine model of sepsis results in improved outcomes in male mice (26), through decreased α-granule secretion of inflammatory mediators and reduced mobilization of P-selectin to the plasma membrane of platelets (26). We have shown that by blocking specific signaling pathways in platelets, we can regulate inflammation without compromising platelet functions. However, P2Y_1_ deficiency did not alter inflammation levels or lung injury in a murine model of sepsis in male mice. However, there are sex-related differences in how platelet respond to activation. In fact, female platelets are more reactive to agonists, especially ADP than male (27, 28) suggesting sex-related differences in ADP- induced platelet activation. Sex-related differences in the expression of P2Y_1_ or P2Y_12_ have been shown to vary within sexes in the murine brain (29) but information about the expression of these receptors in other organs is limited.

In this study, we aim to investigate whether either deficiency or blocking the ADP-receptors P2Y_1_ or P2Y_12_ alters inflammation levels in sepsis in a sex-specific manner. To achieve this aim, we used a well-recognized murine model of sepsis (cecal ligation and double puncture) and compared male and female mice upon P2Y_1_ or P2Y_12_ deficiency or blockade. Our data show that P2Y_12_ but not P2Y_1_ deficiency, decreased the activity of MPO in lungs and kidneys, platelet-leukocyte interaction and platelet activation in male but not female mice. Either P2Y_12_ or P2Y_1_ blockade could decrease activity of MPO in lungs and kidneys and platelet-leukocyte interaction in male mice. On the other hand, P2Y_1_ deficiency but not blockade decreased activity of MPO in lungs and platelet-leukocyte interaction in female mice. In human T lymphocytes, blocking P2Y_1_ or P2Y_12_ alters cell growth and secretion *in vitro* in a sex-dependent manner, supporting the data obtained in mice. Thus, drug targeting purinergic signaling appears to be sex-specific and needs to be investigated to determine sex-related targeted therapies in sepsis.

## Materials and methods

### Materials

All reagents were of analytical grade and were obtained from Thermo Fisher Scientific (Waltham, MA) unless stated otherwise. Ficoll-Paque was from GE Healthcare Bio-Sciences AB (Uppsala, SE). FITC-conjugated anti-mouse CD11b (clone M1/70) was obtained from Invitrogen (Waltham, MA). PE-conjugated anti-mouse CD4 (clone GK 1.5), FITC- conjugated anti-mouse CD41 (clone MWreg30) and FITC-conjugated mouse anti p-selectin (clone RB40.34) were obtained from BD Bioscience (San Jose, CA). FITC-conjugated anti-mouse CD14 (clone MEM-18) were obtained from Sigma-Aldrich (St. Louis, MO). PE-conjugated anti-human CD4 (clone OKT4) was purchased from BioLegend (San Diego, CA) and APC- conjugated anti-human CD8 (clone HIT8a) antibodies was obtained from eBioscience (San Diego, CA) Invitrogen (Waltham, MA). Rat IgG2a κ isotype control FITC [clone eBR2a), rat IgG2b K isotype control PE (clone eB149/10H5)] were purchased from eBioscience (San Diego, CA). Ticagrelor and MRS2279 was obtained from TOCRIS (Pittsburgh, PA).

### Animals and treatments

Animal procedures and handling adhered to the National Institutes of Health standards and were approved by the Institutional Animal Care and Use Committee Protocol #A21040 at North Dakota State University (Fargo, ND, USA). Male and female wild-type and P2Y_12_ deficient pathogen-free C57BL/6 mice (weight, 25-30 g; 8-10 weeks of age) were obtained from Schering-Plough Corporation (Kenilworth, NJ)1-4. P2Y1 deficient pathogen-free C57BL/6 male and female mice were generated by subcontract with Lexicon Genetics Inc. (Woodlands, TX) through knockout constructs as described previously (22, 25, 30). Only 8-10-week homozygote animals were included. Male and female mice were housed in a climate-controlled pathogen free environment and given free access to food and water.

The cecal ligation and double puncture (CLP) procedures were performed on under anesthesia with isoflurane (2 ± 4% induction in chamber and 1 ± 2% maintenance in mask) as described previously (26, 31–33). Following midline laparotomy, the cecum was identified, the mesentery trimmed, and the stalk joining the cecum to the large intestine ligated. Care was taken to assure the intestinal continuity was not interrupted. The cecum was punctured twice with a 24-gauge needle on the anti-mesenteric border, stool expressed, and the cecum returned to the abdomen. Sham control animals underwent a laparotomy without ligation or double puncture. Experiments were performed in P2Y_12_, P2Y_1_ KO, and wild-type (WT) male and female mice that were randomly assigned to one of four groups for wild-type or KO: wild-type and KO sham control group (6 animals per group); wild-type and KO undergoing CLP (CLP group, 6 animals per group).

Ticagrelor and MRS2279 were administrated intraperitoneally to WT male and female mice (6 animals per group) with a dose of 30 mg/kg (ticagrelor) (34–36) and 50mg/kg (MRS2279) (37, 38) at the time of surgery. Sham mice received the same doses of Ticagrelor and MRS2279. After the procedure but prior to emergence, sham and CLP mice were fluid-resuscitated (1 ml/mouse sterile saline, subcutaneously).

At 24 hours post-surgery, mice were anesthetized and blood samples were collected by cardiac puncture (10:1 in 3.8% sodium citrate) for hematology studies (Hemavet^®^ Multispecies Hematology System, Drew Scientific, Inc. Oxford, CT). All mice were euthanized by cardiac puncture and exsanguination. Lungs and kidneys were collected and fixed or frozen immediately in liquid nitrogen.

### Myeloperoxidase activity

Lungs and kidneys were homogenized and sonicated. After centrifugation (10,000 for 10 minutes at 4°C), myeloperoxidase (MPO) levels were detected using a MPO calorimetric assay kit (BioVision, USA). The assay was performed as described by the manufacturer. Briefly, perfused lung and kidney tissues were homogenized rapidly on ice upon addition of myeloperoxidase (MPO) Assay buffer and the supernatant collected after centrifugation (10,000 for 10 minutes at 4°C). Resorufin standard and MPO positive control were prepared. Standards, positive controls, and samples were transferred into different wells of the 96-well plate. Reaction mix prepared with MPO assay buffer, MPO peroxidation substrate, and hydrogen peroxidase (0.88M) was added to each well and thoroughly mixed after which fluorescence (Ex/Em=535/587 nm) was kinetically measured at 37°C for 10 minutes. A resorufin standard curve was plotted and the MPO activity of the test samples was calculated.

### Blood urea nitrogen and creatinine measurement

Plasma aliquots from each animal were obtained by blood centrifugation (2,000 x g for 10 minutes) and immediately frozen. Blood urea nitrogen levels were measured using the urea nitrogen (BUN) colorimetric detection kit (ThermoFisher Scientific, USA). The assay was performed as described by the manufacturer. The plasma samples were diluted with deionized water (1:30) prior to use. The urea nitrogen standard (100 mg/dL urea nitrogen) was used. Samples and standards were added to a 96-well plate. Acidic solutions coded as color reagents A and B in the kit were each added to all wells and incubated for 30 minutes, and the absorbance was immediately read at 450 nm using a microplate reader. Plasma creatinine was determined by an enzymatic assay kit (Mouse Creatinine Assay Kit no. 80350, Crystal Chem, Downers Grove, IL). The assay was performed as described by the manufacturer. Samples were added to a 96-well plate and incubated with the sarcosine oxidase solution provided. Following a 5-minute incubation at 37°C, a peroxidase solution was added to the samples and the plate was incubated for 5 minutes. The absorbance was immediately read at 550 nm using a microplate reader.

### Platelet-leukocyte aggregate formation and P-selectin expression in whole blood

Murine blood samples were diluted 1:2 in PBS and incubated with either FITC-conjugated anti-mouse CD11b (dilution 1:100 in PBS) or CD4 (dilution 1:100 in PBS) or CD14 (dilution 1:100 in PBS) and PE-conjugated anti-mouse CD41 (dilution 1:100 in PBS) or with FITC-conjugated anti-mouse P-selectin (dilution 1:100 in PBS) for 20 minutes at 25°C. The reaction was stopped by adding BD FACSTM lysing solution (1:10 in PBS). Samples were kept in the dark and at 4°C prior to analysis. Flow cytometry was performed using Accuri-C6 System and data were analyzed with FlowJo software. Platelet (CD41^+^) and leukocyte (CD11b^+^) or T lymphocyte (CD4^+^) or monocyte (CD14^+^) aggregates were discriminated by forward and side light scatter and identified by their positive staining for PE-CD41 or FITC-CD11b, or FITC-CD4, or FITC-CD14 respectively. Events double positive for PE and FITC were identified aggregates and were recorded as a percentage of a total of 20,000 gated neutrophils or monocytes or T lymphocytes. Gating strategies are shown in **Supplemental Figures 1A–C**.

### Platelet factor 4 and soluble P-selectin measurement

Plasma aliquots from each animal were obtained by centrifugation (2,000g for 10 minutes) of the blood samples and the plasma was immediately frozen. To detect PF4 and soluble p-selectin concentrations, corresponding ELISA kits (Sigma) were used. The assay was performed as described by the manufacturer. Briefly, Standard protein of mouse PF4 and P-selectin were reconstituted and diluted accordingly to provide a standard stock solution. Samples and standard were added to a 96-well plate coated with either anti-human PF4 or anti-P-selectin antibody, covered and incubated at room temperature for 2.5 hours and overnight at 4°C respectively with gentle shaking. The plates were washed and biotinylated mouse P-selectin or biotinylated anti-mouse PF-4 detection antibodies was added to each well and incubated at room temperature for an hour with gentle shaking. After washing, HRP conjugated streptavidin was added to each well and incubated for 45 minutes at room temperature. Then 3,3,5,5’-tetramethylbenzidine (TMB) in buffer solution was added to each well and incubated in the dark at room temperature for 30 minutes. The reaction was stopped by adding stop solution (0.2M sulfuric acid) to each well and the absorbance immediately read at 450nm using a micro-plate reader.

### Thromboxane generation assay

Plasma aliquots from each animal were obtained by blood centrifugation (2,000g for 10 minutes). Samples were used to evaluate thromboxane generation using a TXB_2_ EIA kit from Enzo Life Sciences (catalog no. ADI-901-002). The assay was performed as described by the manufacturer. Briefly, TXB_2_ standard was prepared using Assay Buffer (tris buffered saline solution supplemented with proteins and sodium azide). Standards and unknown samples were pipetted into the appropriate wells. Blue conjugate (alkaline phosphatase-conjugated with TXB_2_) and antibody solution (rabbit polyclonal antibody to TXB_2_) was pipetted into each well. The plate was incubated at room temperature for 2 hours and washed using a wash solution (tris buffered saline containing detergents). pNpp substrate solution (p-nitrophenyl phosphate in buffer) was added to each well and incubated at room temperature for 45 minutes. The reaction was stopped using trisodium phosphate in water and the optical density read immediately at 405nm.

### Cytokine measurements

Plasma aliquots from each animal were obtained by blood centrifugation (2,000g for 10 minutes). Levels of RANTES, Il-1β, IL-17, TNF-α, and IFN-γ were determined using the high sensitivity mouse cytokine discovery array 32-plex (Eve Technologies, Calgary, Canada).

### Human peripheral blood mononuclear cell isolation

Human blood was obtained from healthy volunteers following informed consent. The Institutional Review Board of North Dakota State University approved the study (#3735). The age of the donors is 39.4 ± 3.0 for females and 38.5 ± 3.6 for males. Blood was collected in 3.2% sodium citrate vacutainers. Blood was diluted with RPMI1640, added to 10 ml of Ficoll-Paque, and centrifuged at 300 g for 30 minutes at RT. Peripheral blood mononuclear cells (PBMC) were collected from the interphase and washed twice in PBS. Viable cell numbers were determined using Eve Automatic Cell counter (NanoEntek, Walthan, MA). Cells with viability higher than 85% were selected and viability was taken into account when the cell number was calculated. Cells (0.5x10^6^ cells/mL) were then cultured at 37°C and 5% CO_2_ in RPMI 1640 media, fully supplemented with penicillin-streptomycin (each at 0.8 mM) and L-glutamine (2 mM).

### Peripheral blood mononuclear cell culture and treatments

PBMC were co-cultured for 3 days at 37°C in the presence or absence of LPS (1μM). To block the P2Y_12_ signaling pathway *in vitro*, we used Ticagrelor (100μg/mL), a well-established selective P2Y_12_ antagonist, which has been used in multiple *in vitro* studies (32, 33, 39, 40). To block the P2Y_1_ signaling pathway *in vitro*, we used MRS2279 (MRS, 100μg/mL), a well-established selective P2Y_1_ antagonist, which has been used in multiple *in vitro* studies (41–44). Negative control cells received an equivalent amount of vehicle (saline). Three-days after LPS exposure, cells were collected and analyzed using flow cytometry. The supernatant was collected and stored at -20°C prior to analysis.

### CD4^+^ and CD8^+^ cell population

**Figure 1:**
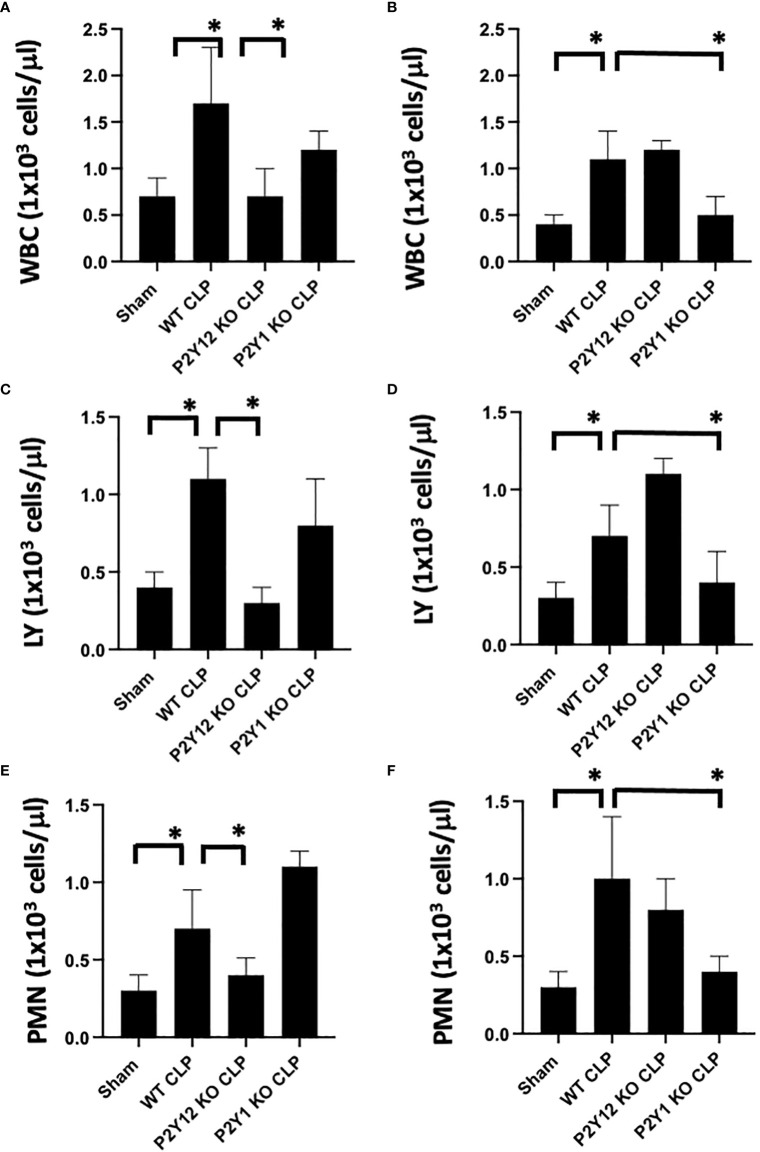
Inflammation-induced elevation in circulating white blood cell counts is decreased in male P2Y_12_ and female P2Y_1_ KO mice. Blood samples were collected by cardiac puncture in 3.8% sodium citrate (10:1), and hematology studies were performed. Graphs show counts of White blood cells (WBC, **A, B**) lymphocytes (LY; **C, D**), and neutrophils (PMN; **E, F**). Sham and CLP in wild-type (WT) and P2Y_12_ KO and P2Y_1_ mice of male **(A, C E)** and female **(B, D, F)** mice. Values are expressed as 1x10^3^cells/μL, mean ± S.E.M. n = 5). (*P < 0.05). No differences between WT, P2Y_12_ KO, and P2Y_1_ KO sham were noted in both male and female mice (data not shown).

CD4 and CD8 phenotyping were measured *via* flow cytometry. Isolated PBMC (0.5 x 10^6^ cells) were incubated with FITC-conjugated anti-human CD4 (1:100 dilution in saline) and PE-conjugated anti-human CD8 antibodies (1:100 dilution in saline) for 1 hour at room temperature. Cells were washed in PBS and kept in PBS at 4°C prior to analysis. Cells were then acquired using Accuri-C6 System and analyzed using the Flow Jo software. The total number of events acquired was 20,000 for each sample. Data are shown as a % of positive events as compared to the total number of events acquired (20,000) (**Figure 1**). Rat IgG2a κ isotype control FITC (clone eBR2a), rat IgG2b κ isotype control PE (clone eB149/10H5) were included as negative isotype controls. Gating strategies are shown now in **Supplemental Figures 1D, E**.

### *TNF-*α measurement

Supernatant from human cell samples were collected by centrifugation (5,000g for 10 minutes) at day 3. TNF-α levels were determined by ELISA (Invitrogen - Waltham, MA). The assay was performed following manufacturer instructions. Briefly, samples and standards were incubated with Biotin-conjugate antibody (anti-human TNF-α polyclonal antibody) at room temperature for 2 hours and washed with assay buffer (PBS with 1% Tween 20, 10% BSA). Then samples were incubated with Streptavidin-HRP for 1 hour at room temperature. The wells were washed using the assay buffer and TMB substrate solution (tetramethyl-benzidine) was added to all wells and incubated at room temperature for 10 minutes. The reaction was stopped using Phosphoric acid (1M) and the absorbance was read immediately at 450 nm using a spectrophotometer.

### IFN- γ measurement

Supernatant from human cell samples were collected by centrifugation (5,000g for 10 minutes) at day 3. IFN-*γ* concentrations were determined by ELISA (Invitrogen - Waltham, MA). The assay was performed following manufacturer instructions. Standards and samples were added to the wells, and incubated overnight at 4°C with gentle shaking. The wells were washed with the wash buffer provided and standards and samples were incubated with biotin conjugate for 1 hour at room temperature. After washing the plate, standards and samples were incubated with streptavidin-HRP for 45 minutes at room temperature. After washing the plate, standards and samples were incubated with TMB substrate solution (tetramethyl-benzidine) in the dark at room temperature for about 30 minutes. The stop solution was added, and the absorbance read immediately at 450 nm.

### Statistical analysis

Differences among groups were statistically analyzed using one-way ANOVA; Bonferroni’s Multiple Comparison Test was used as a *post-hoc* analysis. Each treatment group included four or more experiments (*n* ≥ 4), based on power calculations and work performed previously (45–47). For human cell experiments: each independent experiment was performed using platelets and PBMCs isolated from one donor. PBMCs and platelets from 4 donors were isolated, co-cultured, stimulated and analyzed. Differences among groups were analyzed using a one-way ANOVA test. The analysis was performed in an unpaired fashion. P < 0.05 was considered to be significant. Data are reported as mean ± standard error of the mean (S.E.M.) for each group.

## Results

### Inflammation-induced elevation in circulating white blood cells counts is decreased in male P2Y_12_ and female P2Y_1_ null mice

First, we analyzed the number of circulating white blood cells (WBC), lymphocytes (LY) and neutrophils (PMN) in blood samples (**Figure 1**) from WT, P2Y_1_ KO and P2Y_12_ KO male and female mice. Upon CLP surgery, we observed a significant increase in leukocyte count (white blood cells (A-B), lymphocytes (C-D) and neutrophils (E-F) in septic mice as compared with the Sham group in both male (A-C-E) and female (B-D-F) mice (**Figure 1**, *P*<0.05; male CLP vs male sham or CLP vs female CLP vs female sham). Interestingly, the increase was more pronounced in male septic mice as compared with the female counterpart (**Figure 1**, *P*<0.05; male CLP vs female CLP) for white blood cells, lymphocytes, and neutrophils. However, the sepsis-induced leukocyte count (white blood cells, lymphocytes, and neutrophils) was not increased in the CLP P2Y_12_ KO male mice compared to WT CLP male mice (**Figures 1A, C, E** *P*<0.05; male CLP vs male P2Y_12_ KO CLP) but no change in CLP P2Y_1_ KO male mice was noted (**Figures 1A, C, E**). In female mice, no difference was noted between CLP P2Y_12_ KO mice and WT CLP mice, while leukocyte count (lymphocytes and neutrophils) was not increased in the CLP P2Y_1_ KO female mice compared to WT CLP female (**Figures 1B, D, F**) *P*<0.05; female CLP vs female P2Y_1_ KO CLP. Platelet count did not change in all the groups analyzed (data not shown).

### Sepsis-induced increase of MPO activity in the lungs is reduced in male septic P2Y_12_ and female septic P2Y_1_ KO mice

**Figure 2:**
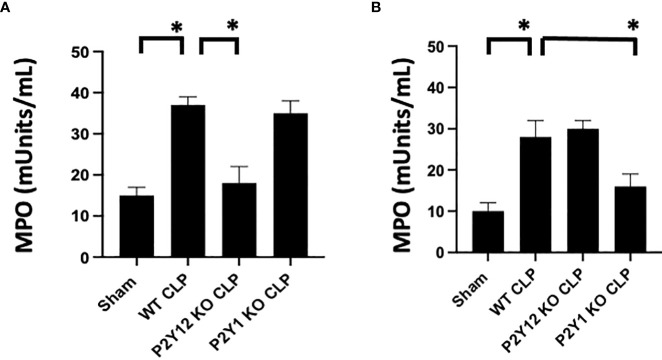
Sepsis-induced increase of activity of MPO in the lungs is reduced in male septic P2Y_12_ and female septic P2Y_1_ KO mice. MPO analysis was performed in lung samples of Sham and CLP in wild-type (WT) and P2Y_12_ KO and P2Y_1_ mice of male **(A)** and female **(B)** mice. Values are expressed as mUnits/mL, mean ± SEM. (*P < 0.05) (n = 5). No differences between WT, P2Y_12_ KO, and P2Y_1_ KO sham were noted in both male and female mice (data not shown).

As the lung is one of the most affected organs during sepsis (48–50), we investigated MPO activity in lung tissue as an indication of neutrophil infiltration in the tissue (**Figure 2**). Lung samples from Sham and CLP mice were analyzed for male and female mice in WT, P2Y_1,_ and P2Y_12_ KO. Although MPO activity was significantly increased in both male (**Figure 2A**) and female (**Figure 2B**) WT mice following CLP as compared with the Sham counterpart (**Figure 2**, *P*<0.05; male CLP vs male sham and female CLP vs female sham), a higher increase in MPO activity was noted in male WT CLP mice as compared with female WT CLP mice (**Figure 2**, *P*<0.05; male CLP vs female CLP). MPO activity was not elevated in the lungs of KO P2Y_12_ CLP mice as compared to the activity in CLP WT mice in males (**Figure 2**. A *P*<0.05; male CLP vs male P2Y_12_ KO CLP), while no difference between WT and P2Y_12_ KO was seen in female mice (**Figure 2B**). These data suggest that P2Y_12_ deficiency alter pulmonary inflammation and inflammatory cell recruitment in male but not in female mice during sepsis. In P2Y_1_ mice, we did not see any difference in MPO activity in male P2Y_1_ KO CLP mice as compared with male WT CLP mice (**Figure 2A**). However, in female mice, MPO activity is significantly reduced in female P2Y_1_ KO CLP compared with WT CLP mice (**Figure 2B**, *P*<0.05; female CLP vs female P2Y_1_ KO CLP). These data suggest that P2Y_1_ deficiency alter sepsis-induced pulmonary inflammation and inflammatory cell recruitment in female mice but not male mice.

### Sepsis induced- platelet activation was decreased in male septic P2Y_12_ and female septic P2Y_1_ KO mice

**Figure 3:**
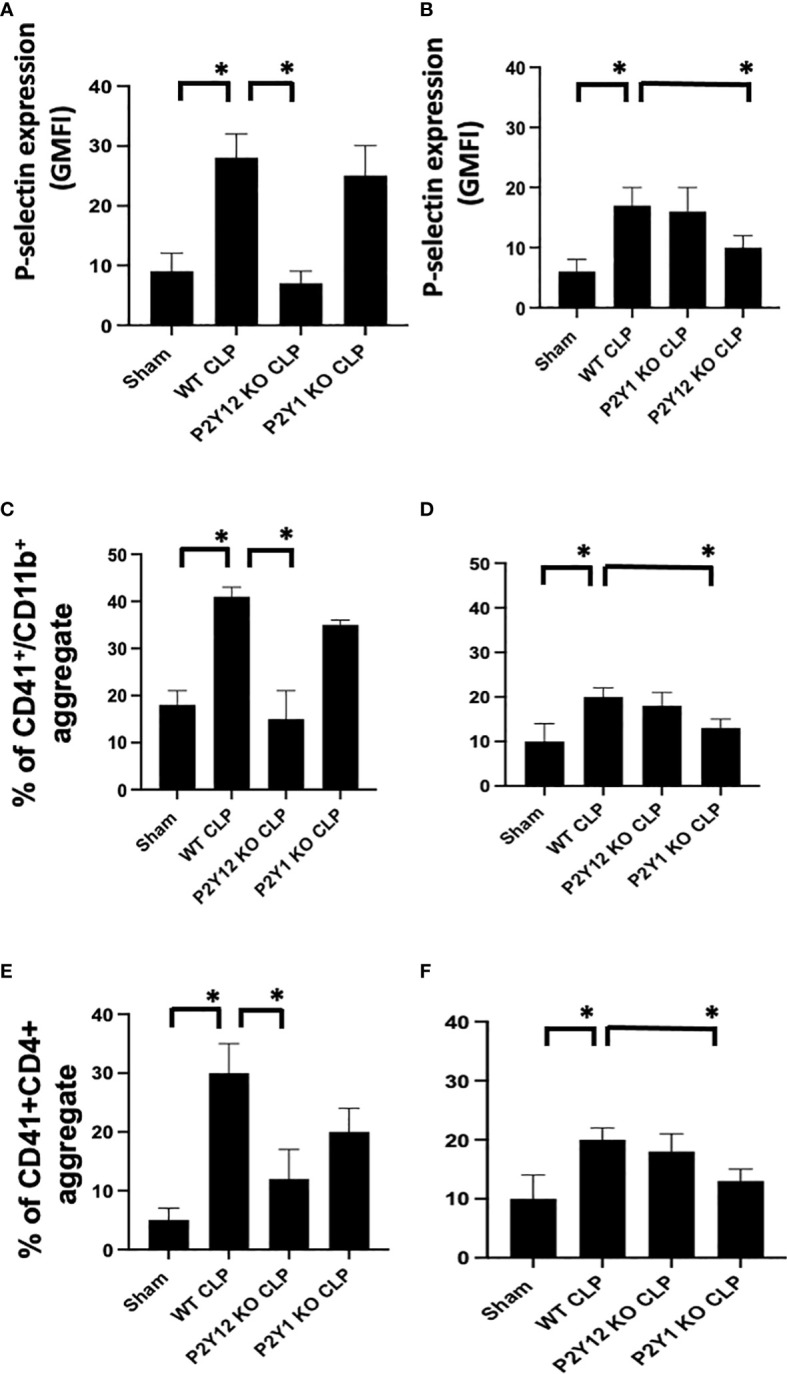
Sepsis-induced platelet activation and CD41+/CD11b or CD41+/CD4+ aggregates were decreased in male septic P2Y_12_ and female septic P2Y_1_ KO mice. Blood samples were collected by cardiac puncture in 3.8% sodium citrate (10:1) **(A, B)** P-selectin expression on the platelet surface was evaluated using flow cytometry in whole blood. Data were collected from WT, P2Y_12_ KO, and P2Y_1_ mice of male **(A)** and female **(B)** mice. Both Sham and CLP were analyzed. (n=6) (*P<0.05) Values are expressed as Geometric Mean of fluorescence intensity (GMFI), mean ± S.E.M No differences between WT, P2Y_12_ KO, and P2Y_1_ KO sham were noted in both male and female mice. **(C, D)** Peripheral blood collected from WT, P2Y_12_ KO, and P2Y_1_ mice of male **(C)** and female **(D)** mice were incubated with antibodies against CD41 (platelet marker) and CD11B (leukocyte marker). Activated leukocytes were gated based on CD11b expression, and cell shape and data were analyzed as a percentage of aggregates expressing both CD41 and CD11b. Values are expressed as the percentage of CD41+/CD11b+ cells, mean ± SEM, n=5). No differences between WT, P2Y_12_ KO, and P2Y_1_ KO sham were noted in both male and female mice. (*P<0.05). **(E, F)** Peripheral blood collected from WT, P2Y_12_ KO, and P2Y_1_ mice of male **(E)** and female **(F)** mice was incubated with antibodies against CD41 (platelet marker) and CD4 (T cell marker). T cells were gated based on CD4 expression and cell shape, and data were analyzed based on the percentage of aggregates that express both CD41 and CD4 (n = 6). No differences between WT, P2Y_12_ KO, and P2Y_1_ KO sham were noted in both male and female mice (data not shown). (*P < 0.05).

To determine whether sepsis-induced platelet activation was sex-dependent or required P2Y_12_ or P2Y_1_ signaling, we measured p-selectin platelet surface expression in septic mice (WT, P2Y_12_ KO CLP and P2Y_1_ KO CLP) for both male (**Figure 3A**) and female (**Figure 3B**). P-selectin was increased in both WT CLP male and female mice as compared with Sham counterparts (**Figure 3**, *P*<0.05; male CLP vs male sham and female CLP vs female sham, *P*<0.05; male CLP vs female CLP). In P2Y_12_ KO CLP mice, sepsis-induced p-selectin values were lower than the WT CLP (**Figure 3A** *P*<0.05, male CLP vs male P2Y_12_ KO CLP) while no difference between WT CLP and P2Y_12_ KO CLP was noted in female mice. Similarly, to what was noted in **Figures 1** and **2**, no difference in p-selectin values was reported between WT CLP and P2Y_1_ KO CLP in male mice, but p-selectin was significantly reduced in P2Y_1_ KO CLP in female mice (**Figure 3B**, *P*<0.05, female CLP vs female P2Y_1_ KO CLP).

### Sepsis induced- platelet-leukocyte aggregate formation in whole blood was reduced in male septic P2Y_12_ and female septic P2Y_1_ KO mice

As platelet-leukocyte interaction is a key step in sepsis, we analyzed platelet-leukocyte aggregate formation in the whole blood and we investigated whether targeting the receptor P2Y_12_ or P2Y_1_ can modulate platelet-leukocyte interaction differently in male and female mice (**Figures 3C, D**). First, we measured interaction between platelets (CD41) and leukocyte (CD11b). The percentage of CD41^+^/CD11b^+^ aggregate was increased in male CLP and female CLP as compared with their Sham counterpart (**Figures 3C, D**, *P*<0.05, male CLP vs male Sham and female CLP vs female sham). Female CLP-induced platelet-leukocyte aggregates was lower than CLP males (**Figures 3C, D**, *P*<0.05, male CLP vs female CLP). In P2Y_12_ KO CLP mice, sepsis-induced platelet-leukocyte aggregates values were lower than the WT CLP (**Figure 3C**; *P*<0.05, male CLP vs male P2Y_12_ KO CLP) while no difference between WT CLP and P2Y_12_ KO CLP was noted in female mice (**Figure 3D**). However, no difference in platelet-leukocyte aggregates was reported between WT CLP and P2Y_1_ KO CLP in male mice (**Figure 3C**), but CD41^+^/CD11b^+^ aggregates were significantly reduced in P2Y_1_ KO CLP in female mice (**Figure 3D**, *P*<0.05, female CLP vs female P2Y_1_ KO CLP).

Second, we analyzed the aggregate formation of circulating platelets (CD41^+^) and T helper (CD4^+^) cells. The percent of platelet-CD4 lymphocytes aggregate was increased during sepsis as compared with the sham control group in male and female mice (**Figure 3C** *P*<0.05, male CLP vs male Sham and female CLP vs female sham), but in P2Y_12_ KO male mice platelets-CD4^+^ T cells aggregates were diminished, suggesting decreased platelets and CD4^+^ T cells interactions (**Figure 3C** *P*<0.05, male CLP vs male P2Y_12_ KO CLP). In female mice, no change was noted in platelets and CD4^+^ T cells interaction upon P2Y_12_ deficiency. No change in platelets-CD4^+^ T cells aggregate was noted in male CLP P2Y_1_ KO mice as compared with male CLP WT mice. But in female mice, platelets and CD4+ T cells aggregate is reduced in female CLP P2Y_1_ KO mice as compared with female CLP WT mice (**Figure 3C** *P*<0.05, female CLP vs female P2Y_1_ KO CLP). These data suggest that P2Y_12_ and P2Y_1_ deficiency reduce platelet-leukocyte and T cell-platelet interactions in a sex-dependent manner.

### P2Y_12_ or P2Y_1_ antagonism attenuates lung and renal MPO activity in sepsis in a sex-related manner

**Figure 4:**
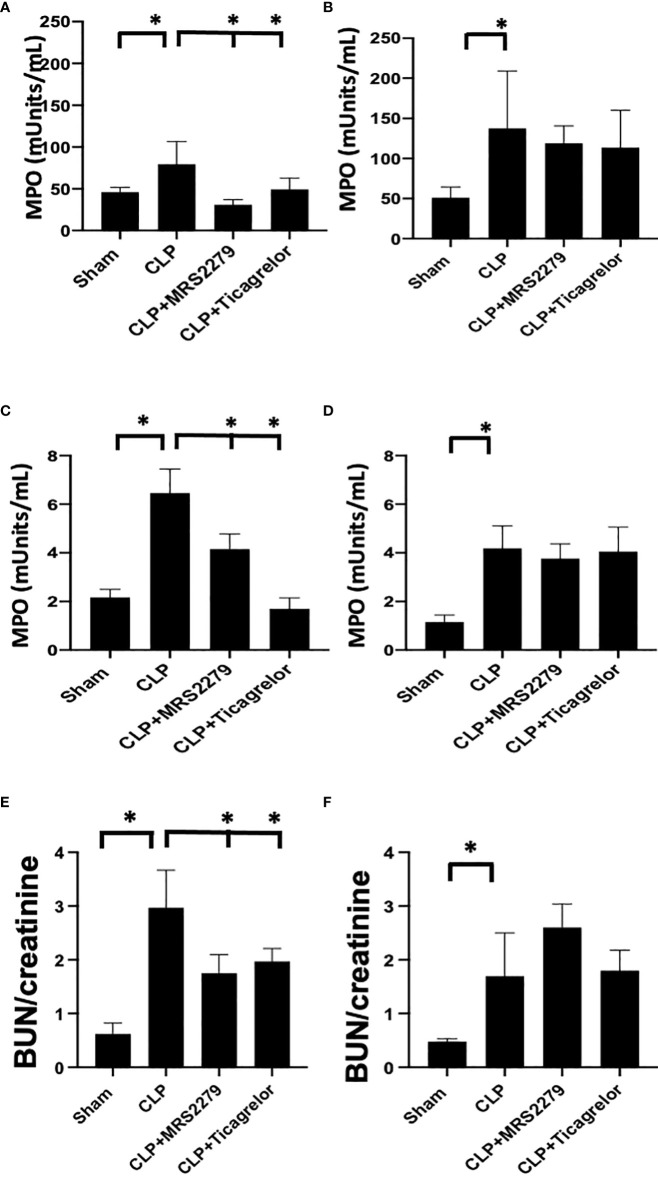
P2Y_12_ or P2Y_1_ antagonism attenuates lung and renal MPO activity in sepsis in a sex-related manner. **(A–D)** MPO analysis was performed in the lung **(A, B)** and kidney **(C, D)** samples of Sham, CLP, MRS2279-treated CLP, and ticagrelor-treated CLP in male **(A, C)** and female mice **(B, D)**. Values are expressed as mUnits/mL, mean ± SEM. (n = 5). (*P < 0.05). **(E, F)** Blood urea nitrogen (BUN)/Creatinine ratios were determined 24 hours post-surgery in plasma samples of Sham, CLP + CLP, MRS2279-treated CLP, and ticagrelor-treated CLP in male **(E)** and female mice **(F)**.

Next, we investigated whether P2Y_12_ or P2Y_1_ antagonism alters sepsis-induced MPO activity similarly to receptor deficiency. Leukocyte trafficking to the lungs and kidneys are among the most affected organs during sepsis (51, 52). At 24 hours post-CLP, there is evidence of both lung and kidney damage associated with increased leukocyte influx (MPO activity) (31, 53, 54). Next, we determined whether blocking the P2Y_12_ or P2Y_1_ receptors alters MPO activity in the lung (**Figures 4A, B**) and kidney (**Figures 4C, D**) of septic male (4A and C) or female (4B and D) mice. **Figure 4A** shows that MPO activity was increased during sepsis in the lung (**Figure 4A**) in male mice as compared with the Sham group (*P*<0.05; male CLP vs male sham). However, when male mice were treated with either MRS2279 or ticagrelor, a significant reduction in MPO was noted in septic male mice as compared with untreated CLP mice (*P*<0.05; CLP vs CLP + MRS2279 or CLP vs CLP + ticagrelor). MPO activity was also increased during sepsis in the lung (**Figure 4B**) in female mice as compared with the Sham group (*P*<0.05; female CLP vs female sham). However, when female mice were treated with either MRS2279 or ticagrelor, no change was noted in MPO levels in the lung in septic female as compared with untreated female CLP (**Figure 4B**). Similarly in kidney samples (**Figure 4C**), when male mice were treated with either MRS2279 or ticagrelor, a significant reduction in MPO was noted in septic mice as compared with untreated mice (*P*<0.05; CLP vs CLP + MRS2279 or CLP vs CLP + ticagrelor). When female mice were treated with either MRS2279 or ticagrelor, no change was noted in MPO levels in the kidney in septic females as compared with untreated female CLP (**Figure 4D**). These data suggest that blocking either P2Y_1_ or P2Y_12_ receptors decreased activity of MPO in the lungs and kidneys of male, but not female mice.

We also investigate a plasma indicator of kidney injury such as blood urea nitrogen (BUN)/creatinine ratios. The ratio was elevated in the CLP group in both males (**Figure 4E**) and females (**Figure 4F**) as compared to their sham control, demonstrating increased levels of blood urea nitrogen BUN/creatinine ratios upon sepsis. Importantly, in male mice (**Figure 4E**), exposure to ticagrelor and MRS2279 resulted in a significant decrease in BUN/creatinine ratios (*P*<0.05; CLP vs CLP + MRS2279 or CLP vs CLP + ticagrelor). No changes were noted in female mice upon treatments (**Figure 4F**).

### P2Y_12_ or P2Y_1_ antagonism alters sepsis-induced platelet activation, and platelet–leukocyte aggregate formation in a sex-specific manner

**Figure 5:**
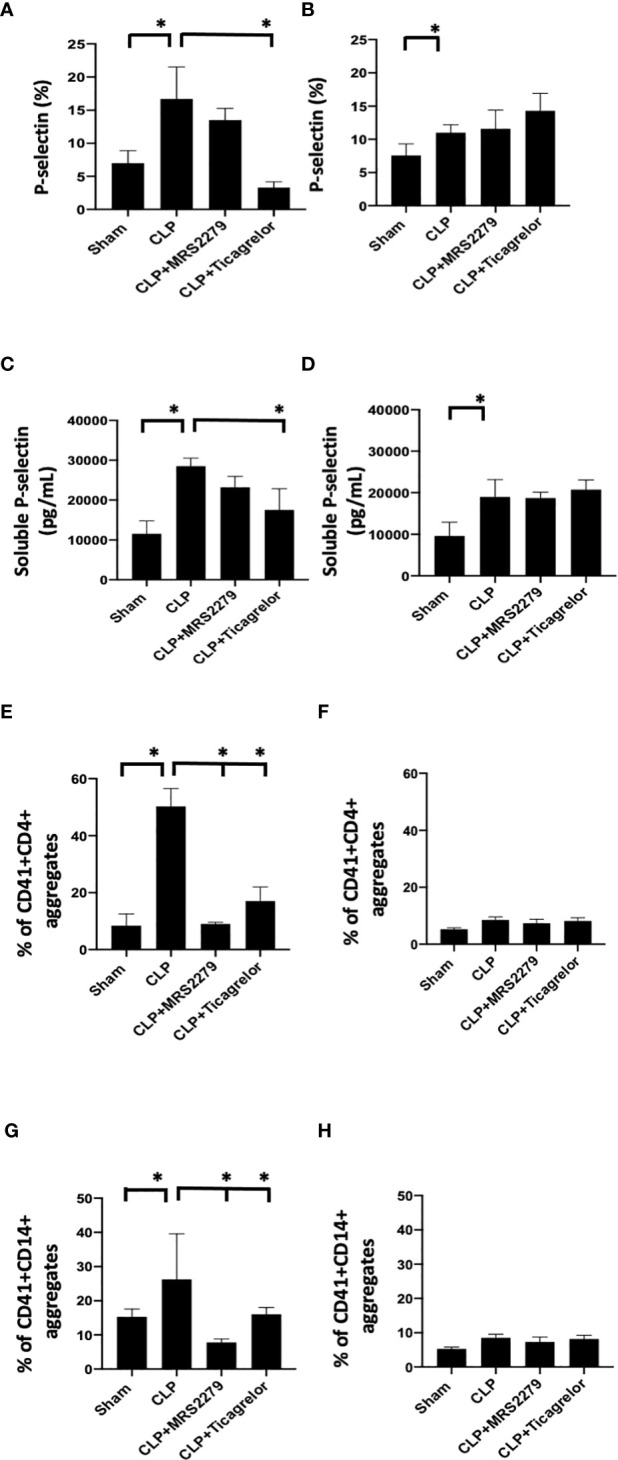
P2Y_12_ or P2Y_1_ antagonism alters sepsis-induced platelet activation, and platelet–leukocyte aggregate formation in a sex-specific manner. **(A, B)**_Blood samples were collected by cardiac puncture in 3.8% sodium citrate (10:1) P-selectin expression on platelet surface was evaluated using flow cytometry in whole blood. Data were collected from Sham and CLP in male **(A)** and female mice **(B)**. Mice were untreated or treated with MRS (50mg/kg) or ticagrelor (30 mg/kg). Values are expressed as % of platelets positive to p-selectin, mean ± S.E.M. (*P < 0.05). **(C, D)** Soluble p-selectin was analyzed in the plasma collected from Sham and CLP male **(C)** and female **(D)** mice using an ELISA kit. Mice were untreated or treated with MRS (10mg/kg) or ticagrelor (10mg/kg). Values are expressed as pg/mL, mean ± S.E.M. (*P < 0.05). **(E-H)** Samples were collected from Sham and CLP male **(E–G)** and female **(F–H)** mice. Samples were incubated with antibodies against CD41 (platelet marker) and CD4 (T cell marker, **E, F**) or CD14 (monocyte marker, **G, H**). T cells or monocytes were gated based on CD4 or CD14 expression respectively and cell shape. Data were analyzed based on the percentage of aggregates that express both CD41 and CD4 **(E, F)** or CD14 **(G, H)**. Values are expressed as the percentage of CD41+/CD11b+ cells, mean ± SEM n = 7).

We investigated whether P2Y_12_ or P2Y_1_ antagonism can influence platelet activation in male and female mice during sepsis. We analyzed P-selectin expression on the surface of circulating platelets (**Figures 5A, B**) and the levels of soluble p-selectin in the plasma (**Figures 5C, D**). In both male (**Figure 5A**) and female (**Figure 5B**) mice, sepsis augmented p-selectin expression on platelet surface as compared with their Sham control (*P*<0.05; male Sham vs male CLP or female Sham vs female CLP). P-selectin expression was higher in septic males as compared with septic female mice (*P*<0.05; male CLP vs female CLP). In male mice, treatment with MRS2279 p-selectin surface expression was not changed in response to CLP, while ticagrelor treatment significantly prevented the sepsis induced elevation of p-selectin surface expression as compared with untreated CLP (*P*<0.05; CLP vs CLP + ticagrelor). In female mice, no change was noted in p-selectin expression upon treatment with either MRS2279 or ticagrelor as compared with untreated CLP (**Figure 5B**). Similar results were noted for soluble p-selectin in the plasma of septic mice. (**Figures 5C, D**). Again, soluble p-selectin was higher in untreated septic male mice as compared with septic female mice (*P*<0.05; male CLP vs female CLP). In male mice, treatment with MRS2279 or ticagrelor lowered sepsis-elevated soluble p-selectin as compared with untreated CLP (**Figure 5C**; *P*<0.05; CLP vs CLP + MRS2279; CLP vs CLP + ticagrelor). In female mice, no change was noted in p-selectin expression upon treatment with either MRS2279 or ticagrelor as compared with untreated CLP (**Figure 5D**).

Aggregates of platelets and other immune cells have been observed in peripheral whole blood during other diseases [22, 23], including sepsis (26, 31). Hence, we investigated the aggregates of platelets and CD4+ T cells (**Figures 5E, F**) or CD14+ cells (**Figures 5G, H**) circulating in the whole blood of sham control, CLP mice, and CLP mice treated with MRS2179 or ticagrelor in both male and female (**Figure 5**). Aggregates were analyzed using flow cytometry. CD4+ or CD14+ cells were gated, and the percent of aggregates as events positive for both CD41 and CD4 **Figures 5E, F**) or CD41 and CD14 (**Figures 5G, H**) respectively was determined. Platelet-CD4+ T cell aggregate formation was increased during sepsis as compared with the sham control group in both male and female mice (**Figures 5E, F**, *P*<0.05; male Sham vs male CLP, female Sham vs female CLP). The % of platelet-CD4+ T cell aggregate was significantly higher in male CLP mice as compared with CLP female mice (*P*<0.05; male CLP vs female CLP). Treatment with either MRS2279 or ticagrelor dramatically diminished aggregate formation in male mice (**Figure 5E**; *P*<0.05; CLP vs CLP + MRS2279; CLP vs CLP + ticagrelor), suggesting that blockade of either P2Y_12_ or P2Y_1_ signaling pathway contribute to decrease platelets and CD4+ T cells interactions in male mice. No change was noted in female mice when either P2Y_12_ or P2Y_1_ were blocked (**Figure 5H**).

### P2Y_12_ or P2Y_1_ antagonism decreased sepsis-induced platelet secretion in both male and female mice

**Figure 6:**
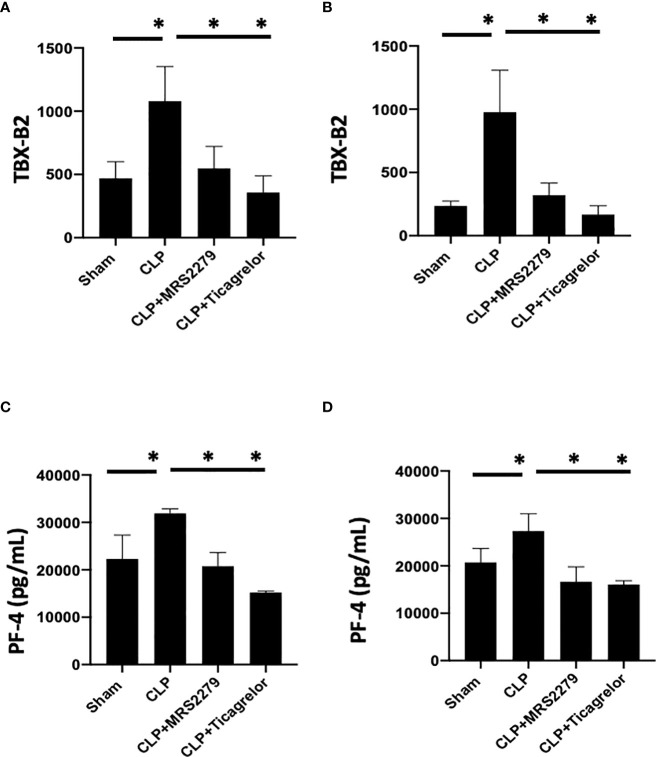
P2Y_12_ or P2Y_1_ antagonism decreased sepsis-induced platelet secretion in both male and female mice. Plasma levels of Thromboxane (TBX-B2) **(A, B)** or PF4 **(C, D)** were evaluated in female (left) and male (right) mice that underwent Sham or CLP surgery. Mice were treated with MRS2279 (MRS) or ticagrelor. Values are expressed as pmol/mL (*p < 0.05, n = 6).

We and other groups have previously observed an increase in platelet secretion during sepsis in animal models and patient samples (26, 31, 55, 56). Hence, we determined platelet secretion in septic male and female mice with and without blockade of either P2Y_12_ or P2Y_1_ signaling pathway (**Figure 6**). We measured plasma levels of platelet-factor 4 (PF-4) and Thromboxane (TBX-B2). As previously observed, both PF-4 and Thromboxane were elevated in male or female septic mice as compared with Sham control (**Figure 6**) *P*<0.05; Sham vs CLP in male and female). Interestingly, in contrast with the data obtained in KO mice (**Figure 4**), levels of both PF-4 and Thromboxane upon sepsis were comparable between male and female mice. Septic-induced Thromboxane increase was not noted in CLP male mice when mice were treated with either MRS2279 or ticagrelor as compared with untreated male CLP mice (**Figure 6A**, *P*<0.05; CLP vs CLP + MRS2279; CLP vs CLP + ticagrelor). Similar data were noted in female mice (**Figure 6B**), where either MRS2279 or ticagrelor treatment prevented the sepsis- elevated level of Thromboxane (*P*<0.05; CLP vs CLP + MRS2279; CLP vs CLP + ticagrelor). Similarly, PF-4 levels were not elevated in CLP male mice when mice were treated with either MRS2279 or ticagrelor as compared with untreated male CLP mice (**Figure 6C**; *P*<0.05; CLP vs CLP + MRS2279; CLP vs CLP + ticagrelor). Similar data were noted in female mice, where either MRS2279 or ticagrelor treatment prevented the elevated level of PF-4 (**Figure 6D**; *P*<0.05; CLP vs CLP + MRS2279; CLP vs CLP + ticagrelor). These data suggest that blocking either P2Y_12_ or P2Y_1_ signaling pathway prevents sepsis-elevated platelet secretion in a sex-independent manner.

### P2Y_12_ or P2Y_1_ antagonism selectively alter cytokine levels in the plasma in a sex-specific manner

**Figure 7:**
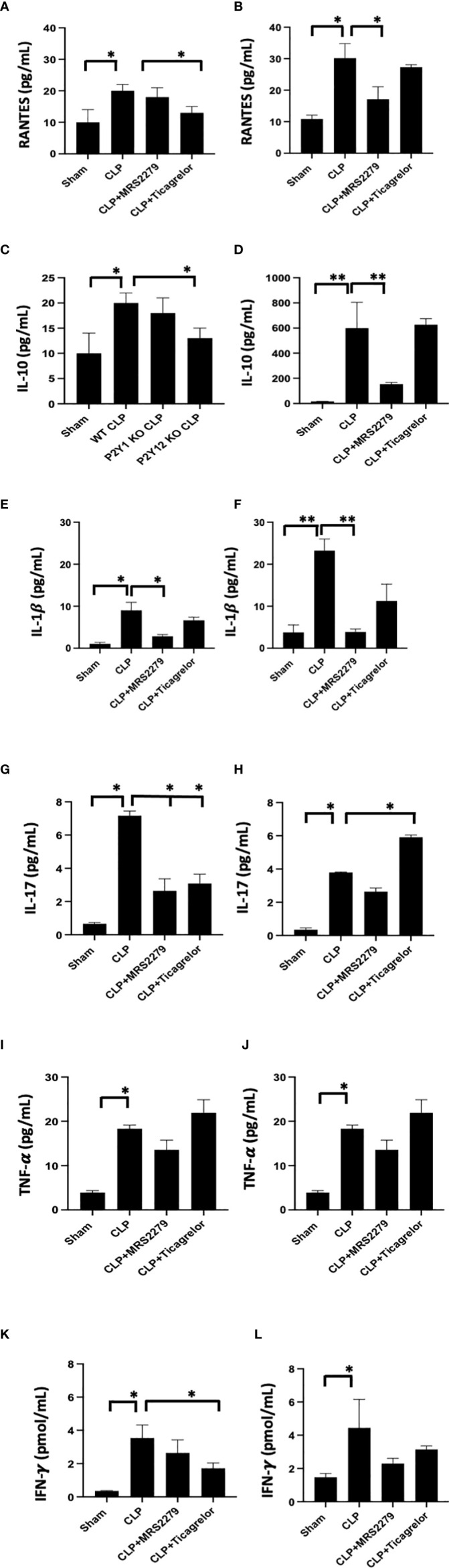
P2Y12 or P2Y1 antagonism selectively alters cytokine plasma levels in a sex-specific manner. Plasma samples obtained from each animal were used for detection levels of RANTES **(A, B)**, IL-10 **(C, D)**, IL-1β **(E, F)**, IL-17 **(G, H)**, TNF-α **(I,J)**, and IFN-γ **(K, L)**. Both Sham and CLP samples were analyzed for male and female mice. Mice were treated with MRS2279 (MRS) or ticagrelor. Values are expressed as pg/mL (*P < 0.05; **P < 0.01; n = 5).

As an increase in circulating cytokines has been observed in patient samples (49, 57) and it has been related to the severity of the disease (58, 59), we investigated levels of RANTES, IL-10, IL-1β, IL-17, TNF-α and IFN-γ in plasma samples (**Figure 7**). As expected, all cytokines’ levels were significantly higher in septic mice as compared with their Sham counterpart for males (**Figures 7A, C, E, G, I, K**) and female (**Figures 7B, D, F, H, J, L**) mice.

For male mice, the sepsis-induced increase in RANTES plasma levels was decreased in mice treated with ticagrelor (**Figure7A**, *P*<0.05; CLP vs CLP + ticagrelor), while no change was noted when septic male mice were exposed to MRS2279. On the other hand, in female mice, the sepsis-induced increase in RANTES plasma levels was decreased when mice were exposed to MRS2279 (**Figure 7B**, *P*<0.05; CLP vs CLP MRS2279), while no change between septic mice was noted when mice were exposed to ticagrelor. Similar data were observed for IL-10 (**Figures 7C, D**) and IL-1β (E-F).

On the contrary, IL-17 levels were non increased in septic male mice treated with either ticagrelor or MRS2279 (**Figure 7G**, *P*<0.05; CLP vs CLP MRS2279 and CLP vs CLP + ticagrelor). On the other hand, in female mice, the sepsis-induced increase in IL-1β plasma levels was noted while mice were exposed to MRS2279 (**Figure 7F**, *P*<0.05; CLP vs CLP MRS2279), while an increase in IL-17 was noted in septic female mice exposed to ticagrelor as compared with septic untreated female mice (**Figure 7H**, *P*<0.05; CLP vs CLP MRS2279 and CLP vs CLP + ticagrelor).

No change was noted in both male and female mice in TNF-α levels in any of the treated groups as compared with septic males and females (**Figures 7I, J**).

Similarly, for male mice, the sepsis-induced increase in IFN-γ plasma levels were noted while mice were exposed to ticagrelor (**Figure 7K**, *P*<0.05; CLP vs CLP + ticagrelor), while no change between septic mice was noted when mice were exposed to MRS2279. On the other hand, in female mice, no change between treated septic mice and their treated counterpart was noted (**Figure 7L**).

### P2Y_1_ antagonism selectively alters CD4 and CD8 cell populations in female but not male human PBMCs

**Figure 8:**
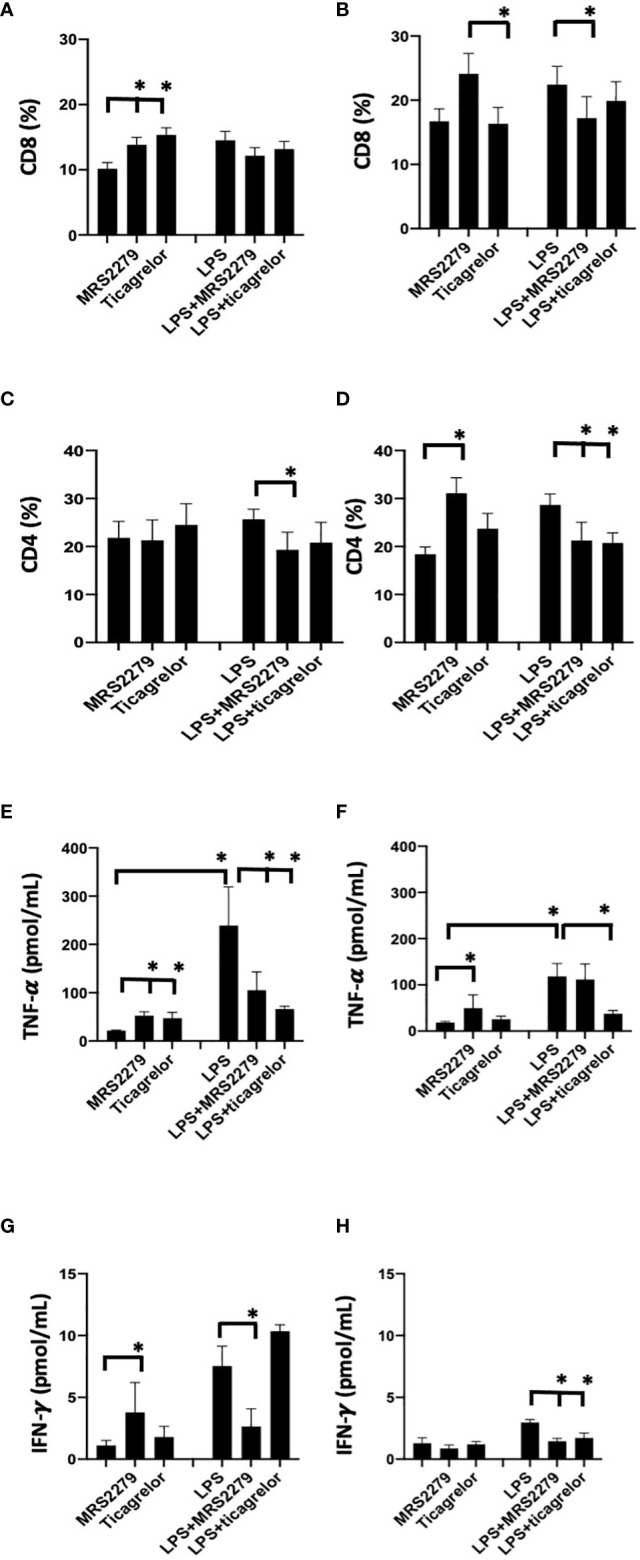
P2Y12 or P2Y1 antagonism selectively alter CD4 and CD8 cell populations and cytokine secretion in human PBMCs. Cells isolated from female (black) and male (white) donors were stimulated with LPS (right panel) or left unstimulated (left panel) for 72 h. Cells isolated from male **(A-C-E-G)** and female **(B-D-F-H)** donors were stimulated with LPS (right panel) or left unstimulated (left panel) for 72 hours. Unstimulated cells were cultured without stimuli. Cells were exposed MRS2279 (100μg/mL) or ticagrelor (100μg/mL). Negative control did not receive any treatment. Cell populations positive to CD8 **(A-B)** or CD4 **(C-D)** were determined using flow cytometry. Data are expressed as % of expression ± S.E.M. (*p < 0.05, n=6). Cytokine levels in the culture supernatants were determined for TNF-alpha **(E-F)** and IFN-γ **(G-H)**. The groups analyzed were: negative control, MR2279-treated, and ticagrelor-treated cells. Cells were stimulated with LPS (right panel) or left unstimulated (left panel) for 72 h. Values are expressed in pg/ml; means ± S.E.M. are plotted (*p < 0.05, n = 6).

To determine whether the sex-specific effects of purinergic signaling blockage that we noted in mice during sepsis is also observed in human cells, we investigated whether blocking P2Y_1_ or P2Y_12_ signaling pathways influence CD4 and CD8 differentiation when PBMCs cells are stimulated with LPS (**Figure 8**). PBMCs were isolated from male and female donors and P2Y_1_ or P2Y_12_ signaling pathways were blocked using MRS2179 and ticagrelor respectively (**Figure 8**, 100μg/mL). PBMCs were incubated with LPS (1μM) for 72 hours. Percentage of CD4 and CD8 cells (**Figures 8A, B**) was determined using flow cytometry and cytokine secretion was determined by ELISA (**Figures 8C, D**).

In PBMCs isolated from male donors, in unstimulated PBMCs the % of CD8+ cells increased in unstimulated cells when treated with either MRS2179 or ticagrelor as compared with untreated unstimulated cells (**Figure 8A**, *P*<0.05; Unstim vs Unstim + MRS2279; Unstim vs Unstim + ticagrelor). No changes were noted in LPS-stimulated PBMCs isolated from male donors. Interestingly in unstimulated PBMC from female subjects, exposure to MRS2279 increased the % of CD8+ cells as compared with untreated cells (**Figure 8B**, *P*<0.05; Unstim vs Unstim + MRS2279; LPS vs LPS + MRS2279). No change was noted when unstimulated cells from female donors were incubated with ticagrelor (**Figure 8B**). Similar results were noted in LPS-stimulated PBMC from female donors (**Figure 8B**). Indeed, exposure to MRS2279 increased the % of CD8+ cells as compared with untreated cells (**Figure 8B**, *P*<0.05; LPS vs LPS + MRS2279). No change was noted when LPS-stimulated cells from female donors were incubated with ticagrelor (**Figure 8B**).

In PBMC isolated from male donors, no change was noted in the CD4+ population in unstimulated PBMCs cells when the cells were incubated with MRS2279 or ticagrelor (**Figure 8C**). However, in LPS-stimulated male PBMCs, the % of CD4+ cells decreased in LPS-stimulated cells when treated with MRS2179 but not ticagrelor compared with untreated LPS-stimulated cells (**Figure 8C**, *P*<0.05; LPS vs LPS + MRS2279). When female PBMCs were incubated with MRS2279, the % of CD4+ cells increased in unstimulated cells as compared with untreated cells (**Figure 8D**, *P*<0.05; Unstim vs Unstim + MRS2279). In contrast, in LPS-stimulated PBMCs the % of CD4+ cells decreased in LPS-stimulated cells when treated with either MRS2179 or ticagrelor as compared with untreated LPS-stimulated cells (**Figure 8D**, *P*<0.05; LPS vs LPS + MRS2279; LPS vs LPS + ticagrelor). These data indicate that blocking purinergic signaling alters the % of the CD8 and CD4 population depending on LPS stimulation in a sex-specific manner.

### P2Y_1_ antagonism selectively alters IFN-γ and TNF-α levels in female but not male PBMCs

Next, we measured the concentration of TNF-α (**Figure 8C**) and IFN-γ (**Figure 8D**) in the supernatant of PBMCs cultured in the presence and absence of LPS (1μM - 72 hours) (**Figures 8E–H**). In male unstimulated cells, TNF-α content was significantly increased when PBMCs were exposed to either MRS or ticagrelor (**Figure 8E**). In female unstimulated cells, we noted a significant increase TNF-α when cells were treated with MRS, but not ticagrelor (**Figure 8E**, *P*<0.05; Unstim vs Unstim + MRS; Unstim vs Unstim + ticagrelor). In male LPS-stimulated cells, a significant decrease was noted when cells were incubated with either MRS or ticagrelor (**Figure 8E**, *P*<0.05; LPS vs LPS + MRS; LPS vs LPS + ticagrelor). In female LPS-stimulated cells, no change was noted in TNF-α in the supernatant when cells were treated with MRS, but a significant decrease was noted when cells were incubated with ticagrelor (**Figure 8F**, *P*<0.05; LPS vs LPS + ticagrelor). In unstimulated cells from male donors, INF-γ content was significantly increased when PBMCs were exposed to either MRS or ticagrelor (**Figure 8G**, *P*<0.05; Unstim vs Unstim + MRS, Unstim vs Unstim + ticagrelor). In unstimulated cells from female donors, no change was noted in INF-γ content between the supernatant collected from unstimulated cells as compared with unstimulated cells treated with either MRS or ticagrelor (**Figure 8H**). In LPS-stimulated cells from male donors, a significant increase was noted when cells were incubated with MRS but not ticagrelor as compared with the LPS-treated control (**Figure 8G**, *P*<0.05; LPS vs LPS + MRS). In LPS-stimulated cells from female donors, INF-γ was significantly decreased in cells treated with either MRS or ticagrelor as compared with untreated LPS control (**Figure 7H**, *P*<0.05; LPS vs LPS + MRS; LPS vs LPS + ticagrelor).

## Discussion

Sex differences in the morbidity and mortality of sepsis have been observed in animal models and human diseases (4–8). To date, females have shown decreased mortality and organ failure in mice and humans compared to their male counterparts (4, 7). However, the lack of studies comparing both sexes limits our capacity to evaluate the extent of sex-related differences and to determine a sex-specific treatment as a result. Our previous studies revealed that platelets are important in sepsis and blocking purinergic signaling in platelets alters the outcome of sepsis in male mice (23, 26, 31). However, we are aware of sex-related differences in purinergic signaling responses (41, 60–63) and in platelet activation (20). Hence, it is essential to investigate how female mice respond to sepsis and whether blocking purinergic signaling alters platelets’ response in a sex-dependent manner. Our data suggest for the first time that the purinergic receptor P2Y_12_ and P2Y_1_ influence sepsis-induced activity of MPO in lungs and kidneys, circulating cytokines, platelet activation and platelet-leukocyte interaction differently in male and female mice. Hence targeting platelets and the purinergic receptor P2Y_12_ and P2Y_1_ may be an appropriate therapeutic strategy that is sex dependent.

In our previous studies in male mice using a CLP model of sepsis (19, 21), P2Y_12_ but not P2Y_1_ deficiency diminished platelet activation and ameliorated the outcome of sepsis (26). When we compared these data with data obtained from female mice, we noted that P2Y_1_ but not P2Y_12_ deficiency could improve the outcome of sepsis, in terms of neutrophil infiltration in the lungs and kidney, platelet activation, and platelet interaction with other immune cells. Previous data have also shown that purinergic signaling is activated differently in male and female mice (27, 28), and the expression of P2Y_1_ and P2Y_12_ receptor varies between sexes (29). Ticagrelor is currently used as anti-platelet therapy (64–66) and clinical studies have confirmed that the effects of ticagrelor in preventing cardio-vascular diseases in patients were comparable between men and women (67–69), hence a sex-related therapy may not be required for cardiovascular disease treatment. However, other studies identified significant changes between male and female patients suggesting a discrepancy in the literature that needs to be clarified (70, 71). The effects of ticagrelor on inflammatory conditions have been studied in a variety of animal models (35, 72–75), although most of the studies were performed exclusively on male mice (36, 73–75). Moreover, clinical trials investigating the effects of ticagrelor in patients during inflammatory conditions, such as inflammatory factors during myocardial infarction (76) or pneumonia (75) has started but they have not provided definitive answers. So far, our data suggest that targeting P2Y_12_ may not be the most appropriate approach to treating females in sepsis, and more data are required to determine whether there is any sex-specificity.

Interestingly, in male mice, blocking P2Y_1_ shows a decrease in activity of MPO in lungs and kidney and platelet-leukocyte interaction, similarly, to blocking P2Y_12_. This was observed in animal models of other diseases, such as colitis (38), Alzheimer (42) and multiple sclerosis (44) when blocking P2Y_1_ improved the outcome of the disease. However, the data are different from what we observed upon P2Y_1_ deficiency. In previous studies, P2Y_1_ blockade and P2Y_1_ deficiency did show comparable results (38, 77, 78), suggesting that the discrepancy we have observed now could be due to sepsis in general or the CLP model. As previous studies investigating either P2Y_1_ deficiency or antagonism were performed almost exclusively in male mice (37, 38, 44, 77), our experiments were the first to investigate changes in inflammation levels in sepsis in female mice upon P2Y_1_ blockade and compare them with the male counterpart. In this model of sepsis, P2Y_1_ blockade did not improve activity of MPO in lungs and kidneys nor altered platelet interaction with other cells. Surprisingly, this is different from what we observed upon P2Y_1_ deficiency. This discrepancy could be due to the dose of MRS2279 used or the off-target effects of MRS2279. We have previously noted P2Y_12_-independent effects upon P2Y_12_ antagonism *in vivo* (26) and *in vitro* (79, 80) but so far, no studies have reported similar results when targeting P2Y_1_. Moreover, the discrepancy could be due to the fact that the P2Y_1_ antagonist is administered at surgery, while P2Y_1_ deficiency may alter organ and/or tissue during development. However, it is interesting to notice that the data appear to be sex-specific as we noted the opposite in male mice. Sex-related differences in the immune response have been reported, indicating that some sex-related differences may be germline encoded. Sex-related differences in purinergic signaling expression and responses in female mice as compared with male mice have been reported (41, 60–63). Cytokine secretion upon inflammation has shown to vary between sexes (81). Indeed, we have observed that cytokine levels such as RANTES, IL-1β, IL-10, and IL-17 are altered by blocking P2Y_1_ or P2Y_12_ differently in septic male and female mice. Hence, purinergic signaling may regulate cytokine secretion in sepsis in a sex-related manner. Blocking P2Y_12_ or P2Y_1_ has shown to change cytokine secretion in several previous studies (26, 31, 74, 82, 83) but changes between male and female mice have not been measured. It would be interesting to investigate thoroughly whether P2Y_1_ and P2Y_12_ are expressed differently in the immune system of males and females. One study investigated LPS-induced lung injury in female mice that revealed platelet activation and neutrophil infiltration is dependent on P2Y_1_ (41). This is a different sepsis model than the one used in this study, but it may suggest that modulating the P2Y_1_ receptor alters platelet response during LPS-induced inflammation. Furthermore, changes in purinergic signaling may be hormonal-dependent. Indeed, other purinergic receptors such as a P2X7 appeared to be hormonal-dependent (14, 16), hence it would be interesting to determine whether the expression of P2Y_1_ or P2Y_12_ is related to hormone secretion.

To determine whether this sex-specificity of P2Y_1_ and P2Y_12_ activation was observed in human cells, we investigated whether PBMCs respond to purinergic signaling blockade *in vitro* when stimulated to LPS. We have previously investigated P2Y_12_ signaling pathways in human T cells and reported that human T cells express P2Y_12_ and P2Y_12_ receptors and blockade altered T cell proliferation and activation in a stimuli-dependent manner (80). However, no experiments have compared P2Y_1_ or P2Y_12_ blockade in LPS-activated PBMCs obtained from male and female donors. Our data exhibited that PBMCs obtained from male donors blocked by either P2Y_1_ or P2Y_12_ inhibitors similarly altered CD4^+^ and CD8^+^ populations, and these changes depended on whether the cells had been activated with LPS. These observations are supportive of our *in vivo* data, where blocking either P2Y_1_ or P2Y_12_ improved the outcome of sepsis similarly. However, in PBMCs obtained from female donors, the CD8+ population growth was altered only when P2Y_1_ was blocked. This is in line with the data we obtained in septic female P2Y_1_ deficient mice. Data on female mice have shown that P2Y_1_ blockade but not P2Y_12_ could decrease leukocyte chemotaxis and platelet-leukocyte interaction (41). Taken together the data support that blocking P2Y_1_ could be a more effective strategy to modulate the immune response in female mice. In future studies, it will be important to evaluate whether these changes are due to sex-related differences in P2Y_1_ and P2Y_12_ receptor expression. There are studies investigating how P2Y_1_ can regulate CD4+ differentiation *in vivo* and *in vitro*. P2Y_1_ deficiency decreased CD4+ population growth and in particular Th17 differentiation during colonic inflammation (77). *In vitro* experiments in human PBMCs have shown that blocking P2Y_1_ could modulate CD4+ cell activation (84). Indeed, mRNA levels of P2Y_1_ are higher than P2Y_12_ (84). In all the studies, no differentiation between male and female cells was analyzed so more data are required before we can identify the most appropriate therapeutic strategy for treating sepsis in both sexes.

The current study has several limitations. First, we have selected the time point of 24 hours post-CLP. It has been shown that at 24 hours organ injury (such as lungs, kidneys, and heart) (48, 50, 85–87), and cytokine levels increase in blood samples of CLP mice (48, 85) are comparable to that noted in septic patients (49, 57). However, to deepen our understanding of sex-related differences in sepsis, exploring a variety of time points is essential. Second, it is true that sepsis can occur at any age, but infants, people with chronic conditions, people with weakened immune systems, and older adults are at high risk (as stated by the Center for Disease Control and Prevention). Indeed, the incidence of sepsis increases with age and is associated with extremely high mortality rates (88, 89). Here, we have selected 8-10-week-old mice, which is comparable to a fertile adult in humans, however, it would be interesting to investigate older or younger male and female mice. These experiments could also investigate whether hormonal changes could be related to P2Y_1_ and P2Y_12_ expression. Finally, exploring a range of doses for both antagonists will also be an essential future study to determine whether the sex-related effects are dose-dependent.

In conclusion, modulating P2Y_12_ or P2Y_1_ receptors can be effective in improving sepsis outcomes, depending on the sex. Targeting purinergic signaling represents a promising therapy for sepsis and identifying sex-specific purinergic signaling may lead to more sex-related targeted therapies in sepsis.

## Data availability statement

The raw data supporting the conclusions of this article will be made available by the authors, without undue reservation.

## Ethics statement

The Institutional Review Board of North Dakota State University approved the study (#3735). The patients/participants provided their written informed consent to participate in this study. The animal study was reviewed and approved by Institutional Animal Care and Use Committee Protocol #A21040 at North Dakota State University (Fargo, ND, USA).

## Author contributions

EA collected materials, performed experiments, and analyzed data. PE collected materials performed experiments and analyzed data. SA analyzed data and wrote the manuscript. GPD analyzed data and wrote the manuscript. SPK analyzed data and wrote the manuscript. LEK analyzed data and wrote the manuscript. EL designed the research study, analyzed data, and wrote the manuscript. All authors contributed to the article and approved the submitted version.

## Funding

This work was supported by the National Institute of Health, grant AI156627-01 to EL, grant HL155694 to SPK, and by the Defense Threat Reduction Agency (Grant #HDTRA11910012) to LEK.

## Acknowledgments

We would like to thank Jamie Brown and Kimberly Peterson from Lillestol Research LLC (Fargo, ND) for collaborating with us in obtaining the human blood samples.

## Conflict of interest

The authors declare that the research was conducted in the absence of any commercial or financial relationships that could be construed as a potential conflict of interest.

## Publisher’s note

All claims expressed in this article are solely those of the authors and do not necessarily represent those of their affiliated organizations, or those of the publisher, the editors and the reviewers. Any product that may be evaluated in this article, or claim that may be made by its manufacturer, is not guaranteed or endorsed by the publisher.

## Supplementary material

Supplementary Figure 1 Flow cytometric gating strategy. Gating strategies are shown to define **(A)** platelet-CD4+ T cell aggregates, **(B)** platelet-CD41+ cell aggregates **(C)** p-selectin surface expression, and **(D, E)** T cell populations.

## Abbreviations

CLP, cecal ligation and double puncture; IL-1β, interleukin-1 β; MPO, myeloperoxidase; PBMCs, peripheral blood mononuclear cells; PBS, phosphate-buffered saline; PF4, platelet factor 4.
