# Supplementary figures and images for: Sex-related differences in the response of anti-platelet drug therapies targeting purinergic signaling pathways in sepsis
Source: Front Immunol. 2022 Nov 2;13:1015577. doi: 10.3389/fimmu.2022.1015577 (PMC9667743; doi:10.3389/fimmu.2022.1015577)

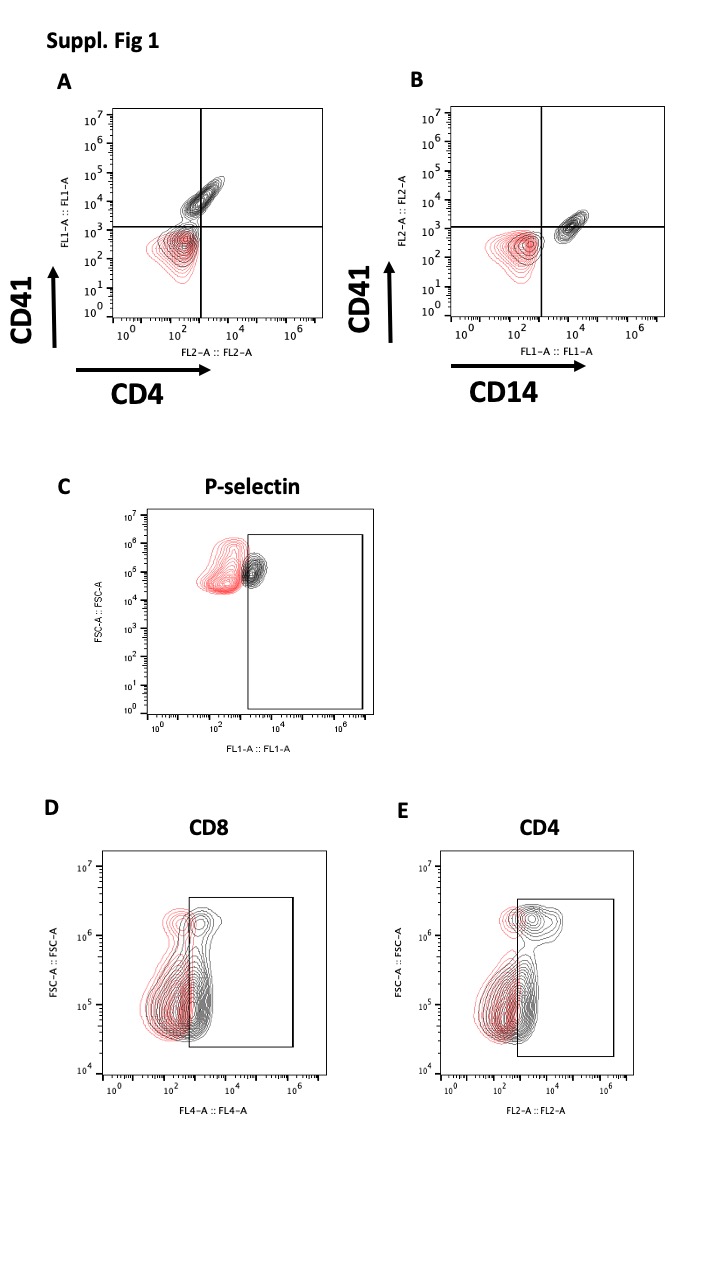

Supplement: Supplementary Figure 1 — Flow cytometric gating strategy. Gating strategies are shown to define (A) platelet-CD4+ T cell aggregates, (B) platelet-CD41+ cell aggregates (C) p-selectin surface expression, and (D, E) T cell populations. [file Image_1.jpeg]
